# The Association between Serological Biomarkers and Primary Sjogren's Syndrome Associated with Peripheral Polyneuropathy

**DOI:** 10.1155/2014/902492

**Published:** 2014-04-13

**Authors:** Che-Wei Hsu, Yu-Jih Su, Wen-Neng Chang, Nai-Wen Tsai, Wen-Chan Chiu, Ben-Chung Cheng, Chih-Min Su, Chi-Ren Huang, Ya-Ting Chang, Cheng-Hsien Lu

**Affiliations:** ^1^Department of Neurology, Chang Gung Memorial Hospital-Kaohsiung Medical Center, Chang Gung University College of Medicine, No. 123, Ta Pei Road, Niao Sung Hsiang, Kaohsiung 833, Taiwan; ^2^Department of Biological Science, National Sun Yat-Sen University, Kaohsiung 80424, Taiwan; ^3^Department of Medicine, Chang Gung Memorial Hospital-Kaohsiung Medical Center, Chang Gung University College of Medicine, Kaohsiung 833, Taiwan; ^4^Department of Emergency Medicine, Chang Gung Memorial Hospital-Kaohsiung Medical Center, Chang Gung University College of Medicine, Kaohsiung 833, Taiwan

## Abstract

*Background and Aim*. The sensitivity and specificity of biomarkers used for predicting peripheral neuropathy of Sjogren's syndrome (SJS) patients remain unsatisfactory. This study aimed to determine the prognostic value of circulating autoantibodies levels in SJS patients with peripheral neuropathy. *Methods*. Two hundred and fifty serological positive (either anti-Ro or anti-La positive) SJS patients' data were collected retrospectively. The titers of autoantibodies, electrophysiology reports, and clinical manifestation were reviewed. *Results*. The prevalence rate of peripheral neuropathy is 7.2% in our study. Regarding classification of peripheral neuropathy, 12 had mixed sensorimotor polyneuropathy, six had cranial neuropathy. After stepwise logistic regression analysis, anti-**β**2 glycoprotein I (a**β**2GP I) and perinuclear anti-neutrophil cytoplasmic antibody (p-ANCA) were significantly associated with peripheral neuropathy in serology positive SJS (*P* = 0.01, *P* = 0.046, resp.). *Conclusion*. The occurrence of peripheral neuropathy among SJS patients is not frequent and easily overlooked. Our study demonstrated that a**β**2GP I and p-ANCA levels may imply the danger of the occurrence of neuropathy in SJS patients, and they can be considered a biomarker that should be added to the panel of conventional autoantibody in SJS patients.

## 1. Background

Peripheral neuropathy remains catastrophic and it could be a fatal complication of Sjogren's syndrome (SJS). The occurrences of neurological system involvement following SJS have reported frequencies between 20 and 60% in different series [1–3]. The detailed classification of peripheral neuropathy was proposed by Gono et al. [1]. According to them, the peripheral nerve system involvement of SJS was divided into three main categories, which were cranial neuropathy, polyneuropathy, and autonomic neuropathy. The pathology of neuropathy varies widely between patients and the clinical manifestations are different [2]. Most neurological system involvement following SJS is peripheral neuropathy [3]. Both the clinical features and pathogenesis were diverse and presented a therapeutic challenge [2–4]. We would like to focus on the category of polyneuropathy of SJS with peripheral nerve system involvement.

This hospital-based study may provide accurate information about the prevalence rate of peripheral nervous system involvement, neuropathy subtypes, their effect on mortality, and their effect on neurological and functional outcome. Because of the possible benefit of therapeutic intervention, there is a need for a better delineation of the potential risk factors and clinical features in this specific group of patients.

Previous studies showed that cryoglobulinemia [5] and anti-Ro [6] are associated with peripheral and central involvement of SJS, respectively. To our knowledge, little clinical researches have focused specifically on biomarkers of SJS complicated with peripheral neuropathy [2–5]. In this study, we analyze clinical features and serological markers to determine potential risk factors that are associated with peripheral neuropathy in primary SJS patients.

## 2. Material and Methods

### 2.1. Study Design

We retrospectively reviewed the medical records, using preexisting standardized evaluation forms, for patients with primary SJS admitted to the Kaohsiung Chang Gung Memorial Hospital (CGMH-KS) between June 2005 and June 2011. CGMH-KS is the largest medical center in southern Taiwan and this facility is a 2482-bed acute-care teaching hospital, which provides both primary and tertiary referral care for patients.

### 2.2. Inclusion and Exclusion Criteria

We retrospectively search our patient database for the ICD-9-CM code of Sjogren's syndrome (710.2) and confirmed with serology markers (either anti-Ro or anti-La) and clinical manifestations between June 1, 2005 and June 1, 2011. Three hundred and two patients with Sjogren's syndrome who had ever required admission were collected. Those Sjogren's syndrome patients who had ever required admission were collected. The diagnostic criteria for primary SJS are according to the revised version of classification criteria of SJS [7], and ocular involvement was confirmed with results of Schirmer's test and Xerostomia was confirmed with either saliva production test or unstimulated salivary flow. Patients excluded were (1) patients whose both anti-Ro and anti-La were negative and (2) patients with secondary SJS (e.g., systemic lupus erythematosus, systemic sclerosis, dermatomyositis, polymyositis, or rheumatoid arthritis). We also excluded those patients with peripheral neuropathy who had had other underlying diseases including diabetes mellitus, cancer, hepatitis, and other autoimmune diseases before Sjogren's syndrome was diagnosed. In this study, patients fulfilled the diagnostic criteria including both electrophysiological study with evidence of peripheral neuropathy and typical clinical manifestations of peripheral neuropathy. Neurologists integrated the neurological manifestations and nerve conduction study's findings during hospitalization. Only 250 patients were finally included in the analysis. The Chang Gung Memorial Hospital's Institutional Review Committee on Human Research had approved the study.

### 2.3. Electrodiagnostic Testing

The NCS/EMG examinations were done by our standard laboratory methods in accordance with the recommended protocol of the American Association of Electrodiagnostic Medicine (AAEM) using a Nicolet Viking Select system (Nicolet Biomedical Inc., Madison, USA). All tests were done under similar temperature conditions in the same room. Skin temperature was maintained at ≥32°C.

### 2.4. Biochemical Analysis

The serum markers of autoantibodies were collected thoroughly from each patient, which include anti-beta 2-glycoprotein I antibodies (a*β*2GP I), anticardiolipin IgM (aCL-IgM), anticardiolipin IgG (aCL-IgG), anti-Ro, anti-La, anti-RNP, anti-Jo-1, anti-Sm, anti-scl-70, anticentromere (anti-cm), perinuclear anti-neutrophil cytoplasmic antibody (p-ANCA), and cytoplasmic anti-neutrophil cytoplasmic antibody (c-ANCA). All of these laboratory data are present with absolute value. We also collect the results of Schirmer's tests (OD: right eye, OS: left eye), saliva production tests (saliva), and unstimulated saliva flow tests (usaliva). The major comorbidities and the clinical symptoms and signs of the index hospitalization were also collected from charts.

### 2.5. Statistical Analysis

Two separate series of statistical analyses were performed. First, baseline clinical data, including gender, clinical manifestations, and biomarkers between those with and those without peripheral neuropathy, were analyzed; categorical variables were assessed by Chi-square test; continuous variables were logarithmically transformed to improve normality and compared using independent *t*-test. Second, stepwise logistic regression was used to evaluate the relationships between risk factors and the presence of peripheral neuropathy, with adjustments for other potential confounding factors. All of the statistical analyses were conducted using the SAS software package, version 9.1 (2002, SAS Statistical Institute, Cary, North Carolina).

## 3. Result 

### 3.1. Demographic Data

The 250 patients included 40 males (age range 32.4–89.6 years; mean age 62.0 years) and 210 females (age range 20.9–92.2 years; mean age 53.6 years). The etiologies of the index hospitalizations, at which time the blood test of anti-Ro or anti-La was checked, among these 250 cases, were listed in Table 1. Among the 250 cases, 83 patients had neurological systems involvement while the other 167 patients did not. Eighty-three cases had neurological systems involvement; peripheral system involvement was found in 34 patients and central system involvement was found in the remaining 49 patients. Among the 34 cases with peripheral nervous system involvement, 18 were peripheral neuropathy, while the other 16 were severe spinal stenosis with radiculopathy. Regarding classification of peripheral neuropathy, 12 had mixed sensorimotor polyneuropathy, six had cranial neuropathy. The common symptom in the 12 cases with sensorimotor polyneuropathy was distal paresthesia associated with weakness on distal parts of four extremities. The levels of the serum a*β*2GP I in patients with or without infection were 1.2 (0–3.7) and 1.7 (0–3.4), respectively (*P* = 0.742). The levels of C-reactive protein (CRP) in those SJS patients with or without peripheral neuropathy were 1.6 (0.75–3.2) and 2.7 (1.2–11.1) (ng/L), respectively (*P* = 0.181). The levels of sedimentation rate (ESR) in those SJS patients with or without peripheral neuropathy were 19 (12–29) and 27 (14–49) (mm/h), respectively (*P* = 0.147).

### 3.2. Serological and Laboratory Data

The levels of autoantibodies are shown in Table 2. The clinical features and laboratory data between the patients with or without polyneuropathy of SJS were compared. Univariate analyses which revealed several biological markers (autoantibodies) were different between two groups, including a*β*2GP I (*P* = 0.001), aCL-IgM (*P* = 0.028), aCL-IgG (*P* = 0.001), anti-scl-70 (*P* = 0.049), p-ANCA (*P* = 0.001), and c-ANCA (*P* = 0.001). All the above variables were used in the stepwise logistic regression models. After analysis of all the aforementioned variables, only a*β*2GP I (*P* = 0.01, 95% confidence interval: 1.015–1.113) and p-ANCA (*P* = 0.046, 95% confidence interval: 1.011–3.167) were independently associated with peripheral neuropathy.

## 4. Discussion

Differences in the relative prevalence of SJS complicated with peripheral neuropathy vary with case determination and inclusion criteria, length of follow-up, underlying conditions, and treatment [4]. Although the frequency of peripheral neuropathy after SJS is variously estimated from 8% to 62% [8, 9], much of these data were based on retrospective studies with variable follow-up. In our study, peripheral neuropathy occurred in 18 out of 250 cases who had SJS (7.2%).

The present study examined the relationship between biomarkers and presence of peripheral neuropathy or not and produced two major findings. First, the prevalence rate of SJS complicated with peripheral neuropathy is low, accounted for 7.2% of the SJS patients. Second, although there are several biomarkers that can be associated with SJS patients, a*β*2GP I and p-ANCA are independently associated with peripheral neuropathy.

Although our study demonstrated that both serum a*β*2GP I and p-ANCA are independently associated with SJS with peripheral neuropathy, our study has several limitations. First, the timing of occurrence of peripheral neuropathy cannot be accessed in this study. Furthermore, peripheral neuropathy could be mixed with sensorimotor polyneuropathy or cranial neuropathy. Both the duration and severity of peripheral neuropathy and its relationship with the level of serum autoantibodies cannot be exactly evaluated in every SJS patient. Our studies involved only one blood sample taken from each patient, and it was evident that there was considerable variation among patients. If such variation followed a standard pattern and temporal relationship, it would reduce variance and improve our ability to predict the prognoses. Second, this was a retrospective analysis and therefore subject to bias of unmeasured factors. It was also not possible to assess the effect of immunosuppressant drugs to prevent the presence of peripheral neuropathy or draw conclusions. Third, patients who had secondary SJS (e.g., systemic lupus erythematosus, systemic sclerosis, dermatomyositis, polymyositis, or rheumatoid arthritis) had been associated with other underlying diseases including diabetes mellitus, cancer, hepatitis, and other autoimmune diseases, and those patients who had only been followed up at outpatient clinic were excluded. Thus, continued uncertainty was present in assessing the incidence of SJS complicated with peripheral neuropathy in nonselected patients. Our findings might underestimate the “true" frequency of peripheral neuropathy associated with the “natural history" of untreated SJS. Finally, the follow-up time was short, which might explain the low incidence in this study and also it did not allow for determination of the recurrence rate of peripheral neuropathy. Thus, continued uncertainty was also present in assessing the incidence of peripheral neuropathy after SJS in nonselected patients. Although the sample size is not large, the numbers of variables considered for the stepwise logistic regression analysis are small. Furthermore, based on the stepwise procedures, only two variables were selected as the important variables predicting peripheral neuropathy. So, the maximum likelihood estimates of the coefficients are valid in the analysis.

Although infection could be a factor associated with elevation of a*β*2GP I [10–13], the levels of a*β*2GP I between infected and noninfected patients were not significantly different (*P* = 0.742). The serum level of a*β*2GP I was found to be associated with thrombosis and fetal loss during pregnancy [14]. When it comes to clinical neurological manifestation, a*β*2GP I links to ischemia events [15, 16] and also a few case reports associated anti-phospholipid antibodies (including a*β*2GP I, aCL-IgM, and aCL-IgG) with autoimmune optic neuropathy [17] and cerebellar ataxia [18–21]. The possible mechanisms of a*β*2GP I in the occurrences of neuropathy among SJS patients in our study included neurotoxic effect and ischemia changes [15, 16, 21].

p-ANCA is a serum autoantibody, which could be detected in vasculitis [22], systemic lupus erythematosus [23], and SJS [24]. Several lines of evidences had demonstrated that p-ANCA is associated with vasculitic polyneuropathy, as we did in this cohort study [25–27]. Whether the p-ANCA is associated with the “Netosis” [28] or not in the pathogenesis of peripheral neuropathy is our focused interest in future study.

In conclusion, the occurrence of peripheral neuropathy among SJS patients is not frequent and easily overlooked. Our study demonstrated that a*β*2GP I and p-ANCA levels may imply the danger of the occurrence of neuropathy in SJS patients, and they can be considered a biomarker that should be added to the panel of conventional autoantibody in SJS patients.

## Figures and Tables

**Table 1:** Clinical comparisons of Sjogren's syndrome with or without peripheral neuropathy.

Sjogren's syndrome	With neuropathy *N* = 18	Without neuropathy *N* = 232
Age	56.3 ± 18.57	54.83 ± 17.77 (*P* = 0.782)
Male/female	4/14	36/196 (*P* = 0.502)
*Discharge diagnosis *		
Peripheral nerve system involvement		
Peripheral neuropathy		
Cranial neuropathy	6	0
Mixed sensorimotor polyneuropathy	12	0
Spinal stenosis/herniated disc	0	16
Central nervous system involvement		
Cerebral infarction or vasculitis	0	16
Movement disorder	0	5
Meningoencephalitis	0	9
Psychiatric problems	0	16
Epilepsy	0	3
Gastrointestinal tract bleeding	0	9
Hepatitis	0	8
Interstitial lung disease	0	6
GYN/OBS related diseases	0	4
Sialadenitis	0	4
Trauma	0	4
Heart diseases	0	4
Endocrinopathy	0	4
Surgery	0	3
Infectious disease	0	77^†^
Vasculopathy/vasculitis	0	13
Metabolic derangement	0	11
Tumor	0	20^*ξ*^

GYN/OBS: gynaecology and obstetrics.

^†^There are 77 cases who suffered from infections, including pulmonary origin in 25, urinary tract infection in 26, and soft tissue infection in 5, and the remaining 21 had infection from other sites.

^*ξ*^The tumors in 10 cases were benign while the other 10 were malignant.

**Table 2:** Laboratory data of primary Sjogren's syndrome with or without peripheral neuropathy.

	With neuropathy *N* = 18	Without neuropathy *N* = 232	All patients *N* = 250	*P* value	Adjusted OR (95% CI)	*P* value
Autoantibody titer on admission						
Anti-*β*2 glycoprotein I	3.6 (2.1–8.2)	1.1 (0–3.5)	1.35 (0–3.68)	0.001	(1.015–1.113)	0.01
Anticardiolipin IgG	6.8 (2.4–9.3)	0 (0–4.1)	0.45 (0–4.3)	0.001		
Anticardiolipin IgM	1.7 (0–3.5)	0 (0–1.4)	0 (0–1.6)	0.028		
Anti-Ro	240.0 (0.7–240.0)	45.6 (0.5–240.0)	56.65 (0.58–240.0)	0.315		
Anti-La	2.9 (0.3–320.0)	0.7 (0.3–15.7)	0.7 (0.3–20.23)	0.183		
Anti-RNP	1.0 (0.6–1.9)	1.0 (0–1.8)	1.0 (0–1.8)	0.286		
anti-Smith IgG	0.2 (0.1–0.5)	0.2 (0–0.4)	0.2 (0–0.4)	0.104		
Anti-scl-70	0.1 (0.1–0.3)	0 (0–0.2)	0.1 (0–0.2)	0.049		
Anti-Jo1	0.1 (0–0.2)	0 (0–0.1)	0 (0-0.1)	0.050		
Anticentromere IgG	0 (0–85.5)	0 (0-0)	0 (0-0)	0.126		
p-ANCA	0.2 (0–0.7)	0 (0-0)	0 (0-0)	0.001	(1.011–3.167)	0.046
c-ANCA	0.3 (0–0.6)	0 (0-0)	0 (0-0)	0.001		
ESR (mm/h)	19 (12–29)	27 (14–49)	24.5 (13.25–46.75)	0.181		
CRP (ng/L)	1.6 (0.75–3.2)	2.7 (1.2–11.1)	2.6 (0.93–8.98)	0.147		
Schirmer's tests on admission						
Right eye	0 (0–1.0)	0.5 (0–10.8)	0 (0–10)	0.05		
Left eye	0 (0–1.3)	0 (0–6.5)	0 (0–5.0)	0.259		
Saliva production tests	0 (0–0.6)	0 (0–2.7)	0 (0–2.39)	0.221		
Unstimulated saliva flow tests	0.1 (0–2.0)	0.2 (0–1.0)	0.16 (0–1.11)	0.994		

p-ANCA: perinuclear anti-neutrophil cytoplasmic antibody; c-ANCA: cytoplasmic anti-neutrophil cytoplasmic antibody; I1QR: interquartile range; CI: confidence interval; CRP: C-reactive protein; ESR: sedimentation rate.
